# Exploring Working Relationships in Mental Health Care via an E-Recovery Portal: Qualitative Study on the Experiences of Service Users and Health Providers

**DOI:** 10.2196/mental.8491

**Published:** 2017-11-14

**Authors:** Monica Strand, Deede Gammon, Lillian Sofie Eng, Cornelia Ruland

**Affiliations:** ^1^ Centre for Shared Decision-Making and Collaborative Care Research Oslo University Hospital Oslo Norway; ^2^ Division of Mental Health and Addiction Department of Psychiatry Blakstad Vestre Viken Hospital Trust Asker Norway; ^3^ Faculty of Medicine University of Oslo Oslo Norway; ^4^ Norwegian Centre for E-Health Research University Hospital of North Norway Tromsø Norway

**Keywords:** eHealth, recovery, mental health, psychiatry, user involvement, empowerment, working relationship, secure email, e-recovery, participatory research

## Abstract

**Background:**

The quality of working relationships between service users and health providers is fundamental in the processes of recovery in mental health. How Internet-based interventions will influence these relationships for persons with long-term care needs, and the measures that can be taken to maintain and enhance working relationships through Internet, is still not well understood.

**Objective:**

The aim of this study was to gain insights into how service users and health providers experience their working relationships when they are offered the option of supplementing ongoing collaboration with an e-recovery portal.

**Methods:**

In this exploratory and descriptive study, an e-recovery portal was used by service users and their health providers in 2 mental health communities in Norway for at least 6 months and at most 12 months (2015-2016). The portal consists of secure messaging, a peer support forum, and a toolbox of resources for working with life domains including status, goals and activities, network map, crisis plan, and exercises. The portal was owned and managed by the service user while health providers could remotely access parts of the service user–generated content. The participants could use the portal in whatever way they wished, to suit their collaboration. Data from 6 focus groups, 17 individual interviews, and an interview with 1 dyad about their experiences of use of the portal over the study period were inductively coded and thematically analyzed.

**Results:**

The thematic analysis resulted in 2 main themes: (1) new relational avenues and (2) out of alignment, illustrated by 8 subthemes. The first main theme is about dyads who reported new and enriching ways of working together through the portal, particularly related to written communication and use of the goal module. Illustrative subthemes are ownership, common ground, goals and direction, and sense of presence and availability. The second main theme illuminates the difficulties that arose when service users’ and health providers’ expectations for portal use were not aligned, and the consequences of not addressing these difficulties. Illustrative subthemes are initiative and responsibility, waiting for the other, feeling overwhelmed, and clarifications and agreements.

**Conclusions:**

The degree to which dyads benefited from using the e-recovery portal appeared to be mainly associated with the degree to which the dyads’ relations were open and flexible before the portal was introduced. For those who experienced frustrations, the portal may have both exposed and added to suboptimal working relationships. Use of the goal module appeared to strengthen the person-centered nature of collaboration. A key question is how health providers balance between enabling service users’ greater control over their care, without relinquishing responsibility for the quality of the working relationship, also when using an e-recovery portal. Implications for implementation are discussed.

## Introduction

Therapeutic alliances, or therapeutic relationships, between service users and health providers in mental health care have repeatedly been found to be of significance for outcomes [1] across a range of diagnoses and treatment settings [2]. Moreover, service users report that their relationship with their health provider is the most important component of care [3] along with their own engagement [4,5]. The study reported here builds on a recovery-oriented approach that uses concepts such as working relationship and partnership to underline the collaboration between health providers and persons in need of long-term mental health treatment and support [6]. The aim of mental health care, and the focus of helping relationships, is to help individuals to live meaningful lives with or without the presence of symptoms [7-9]. Personal recovery is conceptualized as personal processes comprising five dimensions: connectedness to others and the community; hope and optimism about the future; identity building beyond being a patient and toward a positive sense of identity without stigma; meaning in life; and empowerment [10]. Health providers contribute with their professional expertise, whereas service users contribute as the experts in their own lives (eg, their personal values, own needs, and goals) [8]. Accordingly, people are referred to as service users, rather than patients, thus underlining the equality of the roles and expertise (ibid). In recovery-oriented approaches, working relationships focus on fostering service users’ own strengths and resources while developing mutually shared goals, action plans, and outcomes that service users are in charge of, or, through recovery, ultimately take charge of [11,12]. Knowledge about what service users find to be helpful and nonhelpful relationships with service providers is increasing [13,14].

Internet-based interventions are increasingly incorporated into mental health services in ways that can be expected to influence the quality of working relationships [15]. Studies have shown similar or even enhanced working relationships when compared with face-to-face therapies [16,17]. Reported benefits of Internet-based working relationships include facilitation of therapeutic engagement, greater self-disclosure and therapeutic writing, and extensions of the working relationship beyond the active therapy period [18]. Among challenges reported are difficulties in articulation and lack of nonverbal cues, thereby increasing possibilities for misunderstandings [19], as well as a lack of possibilities to respond in real time [20]. Also described is a lack of guidance about how service users and health providers can appropriately engage with each other through the Internet [21]. There is a need for in-depth insights into how such relationships unfold when supported by the Internet [16,20,22], especially when such support is introduced into ongoing care relationships [23,24].

This study examines the use of an Internet-based portal designed to support recovery processes for service users and their ongoing collaboration with their health providers (here referred to as an e-recovery portal). Although research on traditional patient portals has mostly focused on factors such as patient demographics, use and nonuse, and patient engagement and satisfaction [25], attention is increasingly turning to how portals may affect care practices [26]. In this study, we ask: *How do service users and health providers in ongoing mental health care describe their experiences of using an e-recovery portal relative to their working relationships?*

## Methods

### The E-Recovery Portal: ReConnect

ReConnect was designed with service users involved and is described in more depth elsewhere [27]. The portal consists of secure messaging, a peer support forum, and a toolbox of diverse resources that support service users in articulating and working with various aspects of their lives (ie, life domains and associated goals and activities; network map; crisis plan; different exercises related to mindfulness, coping, and symptom management; medication overview; information about user involvement, working relationship, personal recovery, and how to use ReConnect; and links to local activities and service users’ organizations). The portal is owned and managed by the service user while health providers can remotely access parts of the service user–generated content (eg, goals and activities). The portal enables collaboration between the service user and their health provider (here referred to as the dyad) in between or during consultations. Two-hour group and/or individual training sessions were offered in both communities where the study was conducted, so as to accommodate participants’ various schedules and preferences for format. On the basis of the participants’ personal preferences, some dyads participated in a training session together, others participated in group sessions for service users and health providers separately, and a few requested individual training sessions. Local in-real-life ReConnect cafés were held monthly in both communities, at which service users could meet and discuss issues related to their own recovery processes, including the working relationship with their health provider, and portal use. The ReConnect cafés were facilitated by a service user consultant with lived experience of mental health problems.

### Study Methodology and Design

This explorative and descriptive study with a qualitative and participatory design [28-30] studied the use of an e-recovery portal used by service user and health provider dyads in 2 mental health communities in Norway (see the Setting section below). Participants used the portal for at least 6 and at most 12 months (2015-2016). The dyads were told they could use the portal in whatever way they thought might benefit their working relationships and were encouraged to clarify and agree on uses beforehand. The service user consultant was part of the research team throughout all phases of the research process, and participants from the study were invited to give feedback about both the implementation of the portal and the study’s findings as they unfolded (further elaborated below).

### Setting

Norway has universal health care that is publicly funded as part of the national budget through general and earmarked grants. The municipalities are responsible for providing primary health care and social services, while the Regional Health Authorities provide specialist services (eg, hospital wards and district psychiatric centers). As used in this paper, the word “communities” refers to care at primary and specialist levels provided to residents of 2 municipalities in Norway: 1 small community in the North with about 5500 inhabitants within an area of 1493 km^2^ and 1 large community on the outskirts of the capital with about 52,000 inhabitants within an area of 100 km^2^. These were selected for participation to obtain desired contrasts in terms of rural/urban dimensions and access to care. Service users in Norway have at least one specific health provider responsible for the follow-up at each level of care, often a registered nurse, psychologist, and/or psychiatrist. Both communities had expressed commitments to policies promoting eHealth, user involvement, and collaborative practices. The largest community expressed commitments to recovery principles in policy and strategy documents. Local steering committees were established in both communities to ensure that the study and its implementation had local organizational backing.

### Recruitment and Participant Inclusion

Written information about the study, along with verbal presentations individually and in groups, was provided by the research team to multiple health services at both levels of care and to local service user organizations. Interested health providers conveyed the information to service users who they believed might be interested and relevant to the study. In addition, some service users who had heard about the study from other sources requested that their health providers participated with them. Participating service users had to fulfill the following criteria: over 18 years of age, had received mental health services for at least 6 months before inclusion, and had expectations of needing services at least 6 months forward, Internet access with a public key solution for secure electronic identification, and at least one health provider willing to participate in the study. For the health providers, employment in the participating communities and a willingness to participate in the study for at least 6 months with at least one service user were inclusion criteria. Efforts were made to recruit a wide range of participants in terms of age, gender, mental health problems or professional background, and types of ongoing support or workplace. Service users and health providers were invited by the research team, administrators, and/or health providers to take part in focus groups and/or individual interviews about their experiences with use of the portal. For focus groups, participation in the study was the only inclusion criteria. For the individual interviews and interviews with dyads, we intentionally sought participants who had experience of using the e-recovery portal, defined as having logged on to ReConnect >15 times.

### Focus Groups and Interviews

Data were collected using focus groups [31,32] and individual interviews [33], as well as one interview with a dyad who requested that format. Focus groups are suitable for exploring experiences and attitudes among people who cooperate, or have a common frame of reference, and can complement other methods [31]. The focus groups were held at an early stage of implementation so that discussions among participants could also serve to stimulate use and collaboration, a recognized objective of focus groups [34]. The individual interviews were used to facilitate collection of more personal and detailed information (ibid) and were held at a later stage when participants had gained more experience with collaborating through the portal over time. The individual interviews enabled us to explore understandings, perceptions, and constructions of issues that participants have some personal stake in, in line with the objectives of individual interviews (ibid).

The focus groups were conducted with service users and health providers separately to facilitate free-flowing conversations [32]. The interview guide consisted of questions about ReConnect relative to working relationships and recovery processes (see Multimedia Appendix 1). A first draft of the interview guide was discussed with 2 service user consultants who were not part of the research team. In line with the explorative nature of the study, the questions were few and open-ended to stimulate group dialogue [31,32] about the overall study’s 2 main topics: experiences with portal use relative to working relationships and experiences with the role that ReConnect might play in recovery processes. The first of these topics is reported here; the second will be reported in a subsequent paper. Participants were given the opportunity to elaborate on subjects they considered relevant and important. Prompts that could encourage openness, examples, and detail (eg, “That is interesting, can you tell us more about that?”) were used frequently. The focus groups were conducted by MS, who is a trained nurse with clinical experience from the field, and LSE, who was the study’s service user consultant and who had first-hand experience of mental health problems and recovery on both primary and specialist levels of mental health care. The focus groups were conducted after approximately 3 months of participation in the study and lasted for approximately 90 min for the service users and 50 min for the providers.

In the individual interviews and in the 1 dyad interview, we sought more in-depth personal experiences relative to the same topics as the focus groups, also based on semistructured interview guides with open-ended questions (see Multimedia Appendix 2). The individual and the dyad interviews were conducted by MS, with the exception of one individual interview conducted by LSE. These interviews were conducted after 6 to 8 months of participation in the study and lasted for approximately 60 min.

### Thematic Analysis

The focus groups, individual interviews, and the 1 dyad interview were audio-recorded and transcribed verbatim and constitute the entire dataset for this study. Data analysis was aided by use of NVivo software version 11. The data were analyzed by applying a 6-phase thematic analysis for identifying, analyzing, and reporting patterns within the data [35]. The main goal during the analysis was to inductively sort the material into overarching themes and subthemes across the entire dataset, guided by the research question (ibid): *How do service users and health providers in ongoing mental health care describe their experiences of use of an e-recovery portal relative to their working relationships?* MS led the analysis process that entailed the first 3 authors meeting routinely throughout all 6 phases to identify, discuss, and resolve potential differences in, for example, coding and interpretive practices (eg, detail and level of abstraction), thus facilitating multiple perspectives in the process of interpreting the data. In the first phase, authors familiarized themselves with the data, noted initial ideas, and made and discussed preliminary descriptive codes. In the second phase, conducted primarily by MS, relevant extracts of the data (ie, part or all of a sentence, or a small paragraph about 1 particular subject identified in the data related to the research question) were systematically identified and entered into NVivo software version 11 nodes (codes) across the entire dataset. The third phase consisted of collating related codes into preliminary themes and gathering all data relevant to each potential theme. In the fourth phase, the themes were reviewed and adjusted relative to overlaps or inconsistencies both to the coded extracts and the entire dataset. With the goal of generating clear definitions and names for each theme, the fifth phase refined the wording of each theme and the overall story of the analysis. Finally, in the sixth phase, we produced the report by selecting vivid and compelling quotes and to produce a final analysis relating back to the research question. These phases are described sequentially, but in practice, they were conducted as a recursive process (ibid), moving back and forth as needed. Thus, in line with inductive qualitative analysis, the codebook evolved continuously during the analysis [34].

In line with participatory approaches [28-30], participants were invited to give feedback on written and oral tentative summaries of the data through secure messaging, in ReConnect-cafés, or in the individual interviews. This not only facilitated the participants’ contribution to understanding the data but also how to use the e-recovery portal.

The quotes that illuminate identified themes were initially translated from Norwegian to English by DG, a native northern American who is fluent in Norwegian. To minimize the known threats to validity when translating culturally bound expressions [36], the original quotes were kept alongside the translations throughout the development of the manuscript. This enabled all authors to assess the validity of translations, as well as to backtrack to the dataset when context was needed to ensure that the translation captured the quotes’ meaning.

### Ethics

This study was approved by the Regional Committees for Medical and Health Research Ethics in Norway and the Privacy Protection Committees at the participating sites. Participants signed a Web-based consent form with information about the study, which was repeated verbally at the time of the interviews. Service users consented to use ReConnect exclusively for nonemergency purposes and that ordinary channels had to be used for acute needs. Moreover, the participants were given information about security procedures and recommendations for ensuring privacy.

## Results

A total of 14 service users and 17 health providers from both primary and specialist levels of mental health care participated in 6 focus groups, 17 individual interviews, and 1 interview with a dyad.

### The participants

The service users were from 22 to 63 years of age, reporting various mental health diagnoses. The health providers had 1 year to 35 years of clinical experience and represented various professions. Further description of the characteristics of the participants in the different types of interviews is given in Tables 1 and 2.

The thematic analysis resulted in 2 main themes: (1) new relational avenues and (2) out of alignment. These and the 8 identified subthemes are presented below.

### New Relational Avenues

This theme encompasses the ways in which dyads used the options offered by ReConnect to enrich their working relationship and is illustrated through the following subthemes: ownership, common ground, goals and direction, and sense of presence and availability. The process of writing, and uses of the service users’ writings in consultations, is fundamental to this theme and is common to the 4 subthemes.

#### Ownership

In ReConnect, the service users had control over the self-generated content and who had access to it (in contrast to, eg, traditional health records). This shift in locus of control from health providers to service users was described as closely linked to the process of writing that was facilitated by the portal, particularly related to goals, activities, and crisis plans. Health providers, in particular, described how the portal enabled service users to set the agenda for collaboration by describing, in their own words, their situation, priorities, and goals. This in turn strengthened service users’ ownership, or sense of engagement and responsibility, of their recovery processes.

**Table 1:** Characteristics of participants in focus groups.

Characteristics		Service users, N=11		Health providers, N=14	
Age in years, median (range)		45 (22-63)		47 (24-63)	
**Gender, n (%)**					
	Female	11 (100%)		13 (93%)	
	Male	0 (0%)		1 (7%)	
**Site, n (%)**					
	Large community (52,000 inhabitants)	6 (55%)		8 (57%)	
	Small community (5500 inhabitants)	5 (45%)		6 (43%)	
	Primary level	5 (45%)		6 (43%)	
	Specialist level	4 (37%)		8 (57%)	
	Both levels	2 (18%)			
**Diagnosis, n (%)**					
	Depression	6 (55%)			
	Bipolar disorder	2 (18%)			
	Generalized anxiety	2 (18%)			
	Post-traumatic stress disorder	2 (18%)			
	Schizophrenia	1 (9%)			
	Schizoaffective disorder	1 (9%)			
	Phobic anxiety	1 (9%)			
	Panic anxiety	1 (9%)			
	Others	2 (18%)			
Number of diagnosis, median (range)		1 (1-5)			
**Profession, n (%)**					
	Registered nurse			6 (43%)	
	Social worker			3 (21%)	
	Occupational therapist			1 (7%)	
	Interdisciplinary specialist			1 (7%)	
	Priest			1 (7%)	
	Psychologist (clinical)			1 (7%)	
	Psychiatrist			1 (7%)	
Years of clinical experience, median (range)				15 (1-20)	

**Table 2:** Characteristics of participants in individual and dyad interviews.

Characteristics		Service users, N=11		Health providers, N=8	
Age in years, median (range)		45 (24-67)		47 (27-61)	
**Gender, n (%)**					
	Female	11 (100%)		7 (88%)	
	Male	0 (0%)		1 (12%)	
**Site, n (%)**					
	Large community (52,000 inhabitants)	6 (55%)		5 (63%)	
	Small community (5500 inhabitants)	5 (45%)		3 (37)	
	Primary level	7 (64%)		7 (88%)	
	Specialist level	3 (27%)		1 (12%)	
	Both levels	1 (9%)			
**Diagnosis, n (%)**					
	Depression	8 (73%)			
	Generalized anxiety	2 (18%)			
	Post-traumatic stress disorder	4 (36%)			
	Schizophrenia	1 (9%)			
	Schizoaffective disorder	1 (9%)			
	Phobic anxiety	1 (9%)			
	Panic anxiety	4 (36%)			
	Drug addiction	1 (9%)			
	Mania	1 (9%)			
	Others	3 (27%)			
Number of diagnosis, median (range)		1 (1-7)			
**Profession, n (%)**					
	Registered nurse			4 (50%)	
	Occupational therapist			1 (13%)	
	Psychologist (bachelor)			1 (13%)	
	Psychiatrist			1 (13%)	
	Physician			1 (13%)	
Years of clinical experience, median (range)				11.5 (3-20)	

In addition to the processes of writing itself, the use of the service users’ own writings in consultations was described by 1 health provider in a focus group as “enabler of ownership and essential for their [service users’] recovery processes.” Another put it this way:

My client and I have written several crisis plans earlier. And she’s said things about “what are my symptoms” [...]But it’s just recently that she has worked independently with her issues. And put her thoughts into her own words to a much greater degree than before. She’s also done so earlier, but now she has greater ownership to her crisis plan. And that’s important.

Health provider, focus group

Health providers also described how writing appeared to entice service users into taking on a more active role in the relationship and providers could stay more in the background. This is illustrated through the following exchange in a focus group:

A: When it comes to goals...because I’ve been interested in that. I was going to write the action plan. So, it became a kind of win-win situation. The same with the crisis plan which has been very valuable for the service users. And it’s really nice, because they formulate it and write it themselves. So we can kind of sit on the side-lines and...

B: Be lazy...[group laughter]

A: Yes and no. It’s that they work more with it themselves. Their role is a little different when they...

B: Have greater ownership to it.

Health providers, focus group

The opportunity to share what was important and perhaps difficult to share face-to-face further helped service users set the agenda for consultations. It also helped dyads stay focused on what mattered, rather than sliding into conversational habits about nonimportant issues. Health providers reported that receiving messages before consultations helped them prepare and know more of what was expected of them, as shown in the quote below:

It’s sort of nice to have in front of you. To include it in the consultation, if there is something one wants to expand on. Yes, I think it’s good. Just to have an overview of the things the service user had thought about.

Health provider, focus group

Another put it this way:

It’s been...I think a well-ordered way of working on things...A good way of handling our collaboration. In a way, it’s clearer what’s being requested of me.

Health provider, individual interview

#### Common Ground

The secure and asynchronous nature of ReConnect facilitated transferral of clinically relevant information from service users that strengthened the common basis for collaboration in ways not available in ordinary face-to-face consultations. Services users could now describe thoughts and emotions whenever issues arose in real life, rather than having to wait for the next meeting. Because some found it difficult to share thoughts face-to-face, writing helped health providers gain relevant insights.

One service user portrayed the portal as kind of vault where valuables (eg, ruminations) could be safely transferred to the health provider, stored, and dealt with in time. This functioned as a remedy for sleeplessness, as evident from the quote below:

I often write the messages after [health provider] has gone home from work. So, I don’t expect to get an answer until the next day. But for me it’s a way of transferring it to [health provider], thus allowing me to sleep. Instead of ruminating about all this stuff and not getting to sleep.

Service user, dyad interview

The health providers who remotely accessed the service users’ modules (eg, goals and activities) reported that ReConnect yielded qualitatively new and different information than that typically talked about in consultations. In addition, it allowed health providers to monitor progress and tailor their help in a more timely manner, as echoed in the quote below:

It’s much easier to see where my service users are. If some support is needed, if I need to meet with them in-person, or if it’s sufficient to have the appointment that’s already booked, if I need less...sort of knowing how my service user’s everyday life is. I get a grip on this through ReConnect, compared with having a consultation every other week. Then I don’t get so much perspective on the person.

Health provider, individual interview

Despite service users consenting to not using ReConnect for emergency needs, some of their messages could be alarming. One health provider described allowing such messages from 1 service user whom the health provider had worked with over time, before ReConnect. Acceptance of whatever messages came from the service user was described as important to their relationship and common basis for collaboration, despite the dilemmas that this entailed, as shown in the quote below:

I don’t know if I would have been so courageous with others, the way I’ve been with [service user]. To receive and take in what has come [in messages], and dare to let it run its course. Because it’s been a question of living, or not living. It has. But I have also been very clear that this is your choice. I can help you, if you choose to live, but I can’t help you if you choose to die. That’s the way it is.

Health provider, dyad interview

#### Goals and Direction

The mere availability of the goal module appeared to introduce and promote the topic of goals in working relationships, even though actual use of the module itself could vary considerably. Health providers reported working with goals before ReConnect, whereas service users reported that working with goals was new or different after starting with ReConnect. Service users’ descriptions of status in their different life domains, and the formulation of goals from this overview, gave the dyad a greater sense of direction and a basis for monitoring progress. It also helped facilitate insights that service users could experience as more relevant. This is evident from the following quote by a health provider:

She [service user]has most likely come into a new stage in her recovery process, to use it [portal] together with other things that have happened in her life. It [ReConnect] helped her to gain greater insight…she understands herself better. Why she reacts like she does in different situations and what she can do to avoid it. It’s a topic we discuss all the time, but now it’s more like...“Oh, so this is why things are as they are right now.”

Health provider, focus group

An important aspect of working with the modules was that communication in the dyad about issues became more concrete and substantial in ways service users found relevant, as stated by 1 service user below:

So, I feel that I could make things more specific. And have gotten a good start with my providers in ReConnect. That’s probably the most important. That things are more concrete. With my earlier provider, things were really scattered and diffuse [...]Now I’m more receptive to working on things and this tool[portal]has helped, it’s encouraged me to get a grip on things. To get specific. To get an overview. To put things into words, in writing.

Service user, individual interview

In addition to a shift in ownership of the priorities and goals in the working relationship, service users’ resources and knowledge of what helps in everyday life came more into focus.

Such topics ranged from exercising and making friends to getting through the many activities and tasks during Christmas. One of the service users stated:

I think it has actually changed the way we work together. Earlier it’s always been that [health provider] asked me if I’d taken my medications, and then what openings there were in our calendars for my next consultation. Those two issues were what [health provider] seemed mainly preoccupied with. Now with ReConnect we work more on my resources and goals—it can be as simple as managing to get through Christmas. How do I do it? Sub-goals and activities can be: buy the steak, avoid stress, get everything in the house, that type of thing—it was actually very useful to get ideas from another perspective—how to break down the problem...It really helps to break down the problem into smaller pieces.

Service user, focus group

#### Sense of Presence and Availability

Both service users and health providers reported that the portal’s 24/7 availability gave service users a sense of flexibility, extra time, and support in their daily lives. One service user said:

She [health provider] has been really good at giving me feedback [...] like when I can’t sleep, she answers me when she comes to work at 8 o’clock [in the morning], but then it doesn’t wake me up.

Service user, individual interview

Both persons in the dyad described that the opportunities for written communication between consultations facilitated a sense of availability. One service user explained that it was like the health provider was with her in her own living room and that the opportunity to send messages prevented long waiting hours through the night. One of the health providers underlined the importance of having a sense of presence and availability as follows:

It’s sort of that they feel that they are part of something greater, maybe. That there is a connection somewhere out there. Either through the forum, or...maybe their health provider, or someone they can be in touch with. To know someone is there.

Health provider, individual interview

For another health provider, the portal enabled greater flexibility in that the health provider could respond promptly to the needs of service users outside consultations. Although this did not necessarily require more time, service users might experience it that way, as described in the quote below:

To be completely honest, I really think they feel like they get more time with me and feel more appreciated.

Health provider, individual interview

### Out of Alignment

This theme illustrates difficulties that participants described about working together through ReConnect and contains 4 subthemes: initiative and responsibility, waiting for the other, feeling overwhelmed, and clarifications and agreements.

#### Initiative and Responsibility

Service users reported difficulties in taking the initiative and responsibility to work with their health provider through ReConnect. Some did not know exactly what they needed or how to ask for help and reported that they did not want to disturb the health provider, who had an already heavy workload. Although service users acknowledged their own responsibility to take action and to set the agenda in the collaboration through ReConnect, they also described how this could be difficult as follows:

When you’re struggling, at least I find it very difficult to sit down and write things to my helper. I would prefer that my helper would write to me first.

Service user, focus group

Health providers explained their lack of initiative by referring to characteristics of the portal, which was intended to be owned and managed by service users. They also argued that the initiative and responsibility should be with the service users as part of their recovery process. Health providers also expressed concerns that encouraging use might be an added burden for the service users. However, health providers also stated that they did not consider the portal suitable for everyone. One of the health providers said:

Yes, I’ve read some things about user involvement. How it should be. And then you think that maybe it doesn’t work for everyone [...]. I’ve maybe thought of this as being user-controlled, so you [service user] can do more for yourself.

Health provider, individual interview

Mainly, it was the service users’ initiatives that determined the health providers’ activity in the portal, mostly for reading and/or responding to messages. However, some health providers described that they encouraged activities in ReConnect such as working with different modules in the portal, initiating messages, and following up work with goals and activities both online and in consultations. The health providers’ initiatives toward use of ReConnect were highly appreciated and considered essential for successful use by some of the service users. For other health providers, the service users’ expectations to take initiative and responsibility for use were difficult to fulfill, as described below:

I have a feeling that she had somewhat higher expectations on my...that I should have been more active. But...it’s not really the way we work.

Health provider, individual interview

#### Waiting for the Other

For service users, the lack of response from their health providers to their initiatives through ReConnect resulted in feelings of mistrust and not being appreciated. As one service user said,

Just feeling that one is not believed. Feeling not being taken seriously.

Service user, individual interview

Some service users described the work in ReConnect without the support of the health provider as meaningless. One service user reported needing support especially in working with goals, but that the health provider had not responded, as follows:

But then it’s really important that you have your helper on the other end. That you work together in ReConnect with goals for example. I haven’t had that.

Service user, focus group

Health providers expressed awareness of how unmet initiatives and expectations could potentially be harmful for service users. However, difficulties in expressing oneself in writing about complex issues was one health provider’s explanation for not responding to service users’ messages:

I find it difficult to answer in writing, sort of...in some ways...when there are a lot of questions. And she wanted me to say what I think. It gets very difficult. Because it’s the kind of thing that’s best discussed in a dialogue. Verbally. It gets so...when there isn’t any wrong and right, sort of, in what we’re working on. And I can’t do it in writing. This is something that she experienced as a disappointment, and thought, yeah, we should have clarified that ahead of time.

Health provider, individual interview

Some health providers also described frustration over a lack of response to their initiatives to use ReConnect. For one health provider, a lack of response from the other decreased her motivation to take new initiatives:

So...you know I feel that...when that after that long response, and [service user] doesn’t answer. Then the gas sort of goes out of my balloon.

Health provider, focus group

#### Feeling Overwhelmed

Several health providers described different experiences of being overwhelmed when using ReConnect. Frequent messages, ambiguities in how to respond, and a call for being more proactive in the use of ReConnect were found difficult. One health provider elaborates,

...if there are three A4 pages with dense text that I have to go through, then it’s sort of...then I can feel that I don’t measure up sort of. And she might have experienced that she didn’t get the response that she was hoping for.

Health provider, individual interview

Furthermore, therapeutic responses to messages from the service users were found time-consuming, in addition to causing concerns with how the text could be interpreted by the receiver. As one health provider stated:

You’ve got to watch what you write. Many patients can be easily offended. Some patients are really obsessed with details. And it’s not your intention to hurt feeling, or…right. But it can be perceived like that. So that’s why you have to be very careful with how you express yourself. Where the commas, and the periods are. And I experience that as demanding.

Health provider, individual interview

The work with ReConnect came on top of what health providers described as a heavy workload and was difficult for some to balance. During a group interview with health providers, the interviewer conveyed a wish expressed by a service user who would have liked it if the health provider could send a message asking “How are you doing?” now and then. This prompted 1 health provider to burst out that she regretted participating in ReConnect, as shown in the quote below:

So, when I hear that kind of thing I get...I don’t have a problem being nagged at, that doesn’t upset me. But having to go around with other peoples’ issues in my head all the time, I just don’t have the capacity. So, when you said that…you know what? [...]I need to get out of this. That was exactly what I felt.

Health provider, focus group

#### Clarifications and Agreements

As was evident throughout the above 3 subthemes, few dyads reported explicitly addressing expectations or making agreements about how they would use ReConnect. In hindsight, most of the health providers expressed that such discussions would have made the collaboration easier and reduced uncertainties. One health provider stated:

Yes, I think it would have been best to set aside time at the beginning and do things, test it out together. That would probably have contributed to a safer basis for using the portal in a better way.

Health provider, focus group

However, a lack of clarifications and agreements was not necessarily experienced as a problem, as reported by 1 service user below:

No, we have never really had any agreement about how we would work. [...] ReConnect has in a way been an extended arm for me. It’s very seldom [health provider] writes anything on her own initiative. So, it’s me who opens communication.

Service user, individual interview

One health provider underlined a need for recurrent discussions about use and expectations as collaboration progressed and needed adaptations, as shown in the quote below:

Back to the issue of expectations. I think there are, in a way, several layers to the issue. The first is to clarify expectations about response times and such. That’s one thing, but then if a service user is active, that they use exercises, or are working on new goals that you need to be involved in, then it’s important to make new clarifications. What type...where are we now? How do we do this together? So, that you have to take a new round each time.

Health provider, individual interview

## Discussion

### Principal Findings

This study describes service user and health provider experiences in ongoing mental health care with an e-recovery portal as a basis for exploring the potential role it may play in working relationships. The 2 main themes that emerged from our data depict 2 contrasting roles.

#### Exploiting New Relational Avenues

The main theme *new relational avenues* describes how dyads used the e-recovery portal to enrich their working relationship. Mainly described by the health providers, ReConnect strengthened service users’ sense of ownership in their care, largely through the goal module and writing process. The writings from service users also offered health providers broader and more nuanced insights from the service users’ perspectives, and thus a more person-centered basis for working together, also during in-person consultations. ReConnect was described as helping to focus collaboration on the needs and goals that service users considered relevant to their daily lives; for example, a positive Christmas for one’s family. Service users’ 24/7 access to the portal promoted a sense of providers’ presence and availability despite asynchronous, and sometimes lengthy, response times.

In addition to coinciding with other studies showing the potential of e-health technologies in fostering engagement in treatment and care, self-disclosure, and therapeutic writing [18], our study found that use of the goal module, in particular, appeared to boost the person-centered nature of collaboration. Setting the agenda and doing things for oneself is central to recovery-oriented practices [37,38]. However, findings from a review study of care plans indicate that goals and actions are mostly formulated in terms of actions to be taken by providers on behalf of service users [39]. Incorporating support for goal formulation and follow-up into service users’ own portal for collaboration with providers, as done in this study, appears to be a promising way of counterbalancing this. Moreover, the examples of successful uses of the goal module illustrate how service users’ values, preferences, strengths, and resources relevant to their everyday lives came more to the forefront of collaboration. This coincides with recovery-oriented approaches [39] and descriptions of helpful relationships [13]. However, such benefits are not given. Common among dyads reporting positive portal use was that they had health providers who elicited and were responsive to service users’ initiatives and needs.

#### Aligning Expectations and Responsibilities

The main theme *out of alignment* highlights the difficulties that arose when service users’ and health providers’ expectations were not aligned and when the resulting difficulties were not addressed. Nonhelping relationships have earlier been described as impersonal and lacking space for negotiation of the relationship, and the support and treatment provided through it [14]. Although participants were encouraged during the training sessions to discuss beforehand how they would use the portal, few did so explicitly. Those who expressed frustrations, both among service users and health providers, reported expecting initiatives or responses via the portal that the other party failed to fulfill. Service users experienced this as not being taken seriously, whereas some health providers reported losing motivation to use the portal.

Some health providers who neglected to initiate contact with service users via the portal explained this by referring to the information about the study underlining that ReConnect was mainly the service users’ portal that they owned and managed. When service users were uncertain about use, or needed encouragement from their health providers to use the tool, a lack of initiative from health providers brought use to a standstill, which service users described as frustrating. This raises the issue of how health providers can balance between enabling service users’ greater engagement, responsibility, and control of their recovery process, without relinquishing responsibility for the quality of the working relationship in care processes when using e-recovery portals.

The factors collectively grouped under the subtheme *feeling overwhelmed* refer to the experiences of health providers, especially related to well-known difficulties with the use of written communication in a working relationship [19]. Such factors include discomfort in receiving frequent and long messages, service users’ expectations about frequency and content of responses, difficulties in articulating oneself in writing, fear of misunderstandings due to lack of nonverbal cues, and heavy workload. It should be noted that none of the service users reported expecting immediate or therapeutic responses to their messages. Interestingly, health providers’ frustrations over not being able to respond in real time [20] were not reported by service users. Instead, nonsynchronicity was described as enabling them to rest after transferring difficult issues over to the provider, knowing it would be addressed in time.

Although some dyads clarified their understandings about how to use the portal and adjusted accordingly, others did not. Dyads that were enriched by use of ReConnect, despite not explicitly agreeing on how to use the system, appeared to have relationships that were open and adaptable at the outset. For some of those who experienced frustrations, the portal appeared to expose and sometimes reinforce suboptimal working relationships. An earlier study of portal use reported that service users and health providers seek guidance for how to appropriately engage with each other through the portal [21]. Although such guidelines may have helped reduce some of the difficulties the participants experienced in reaching a common understanding in our study, it is not clear that relationships that are nonhelping at the outset will be improved by such guidelines.

### Limitations

Our own involvement in the design of ReConnect, as well as participants’ knowledge of our involvement, poses known risks to the trustworthiness of our findings [40]. We have sought to limit these risks by addressing them repeatedly in the research team throughout the study and by providing thorough and transparent descriptions of context and method. Discussions with participants about our preliminary findings (see Methods section) helped us critique and nuance our evolving interpretations of the data. The conduct of the study as part of ongoing community practices and the inclusion of participants with diverse ages, mental health problems, and professional backgrounds should strengthen the transferability of findings, at least in a Norwegian context. However, the study’s dependability, that is, awareness of the degree to which the data changed over time, could have been discussed by the research team in further detail (ibid). The gender bias toward women, despite concerted efforts to recruit men, is also a limitation to this study.

### Implications for Practice and Further Research

In efforts to ensure that e-recovery portals such as ReConnect enhance rather than undermine the quality of working relationships, some suggestions can be derived from our findings.

Instead of introducing a portal to dyads by saying “use it as you see fit,” as done in this study, more detailed information and recommendations would likely have benefited dyads. Such information would include the advantages and disadvantages that others have experienced with portal use, recommendations for how to clarify mutual expectations (eg, response times, type of content, and how to resolve disagreements about preferences for use), and that agreements for use need to be revisited as parties gain experience with use.

Furthermore, implementation of an e-recovery portal into organizations is probably more likely to be successful if coupled with organizational commitments to recovery principles as described in the literature [6]. This includes training providers in how to foster good working relationships (eg, responsiveness to service users’ initiatives). Although we have yet to test this, we believe such training might be more effective if feedback-informed methods [41] were incorporated into the portal. Without the above dyad clarifications and organizational support, our findings suggest that health providers who are skeptical to using a portal for collaborating with service users should probably refrain from use regardless of the wishes of service users.

Our hypothesis that the quality of preexisting working relationships is the primary determinant of the benefits of an e-recovery portal (rather than the portal itself) needs closer study. If such portals can play an independent role in benefiting or undermining working relationships, then we need to know more about how and by which mechanisms, some of which are suggested by our findings. A key question is how health providers can balance between enabling service users’ greater engagement, responsibility, and control in their own care, without relinquishing responsibility for the quality of the working relationship, also when using e-recovery portals.

Furthermore, we propose that the goal module in particular strengthens person-centered collaboration and is worthy of further study. For example, how does collaboration through the goal module affect providers’ engagement in the service users’ priorities and goals? More knowledge is also needed about gender preferences to ensure that tool and intervention design is inclusive of both genders. Finally, more knowledge is needed about how to optimally leverage the expertise of service user consultants in promoting positive working relationships both online and in real life.

### Conclusions

The degree to which service user-health provider dyads benefited from portal use appeared to be mainly associated with the degree to which the dyads’ relations were open and flexible before the portal was introduced. For those who experienced frustrations, the portal may have both exposed and added to suboptimal working relationships. Use of the goal module, in particular, appeared to strengthen the person-centered nature of collaboration. A key question is how providers balance between enabling service users’ greater control over their own treatment and care, without relinquishing responsibility for the quality of the working relationship, also when using an e-recovery portal.

## Appendix

Multimedia Appendix 1 Interview guide for focus groups.

Multimedia Appendix 2 Interview guide for individual and dyad interviews.
