# Supplementary figures and images for: Exploring Working Relationships in Mental Health Care via an E-Recovery Portal: Qualitative Study on the Experiences of Service Users and Health Providers
Source: JMIR Ment Health. 2017 Nov 14;4(4):e54. doi: 10.2196/mental.8491 (PMC5705858; doi:10.2196/mental.8491)

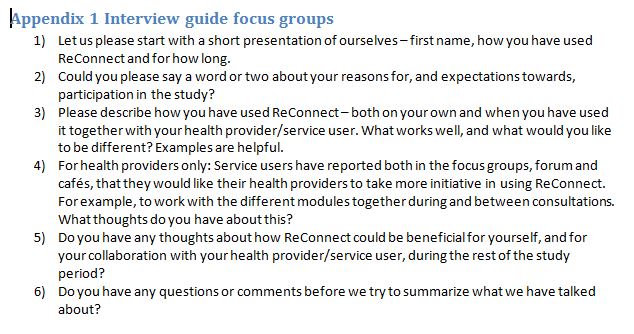

Supplement: Multimedia Appendix 1 [file mental_v4i4e54_app1.JPG]

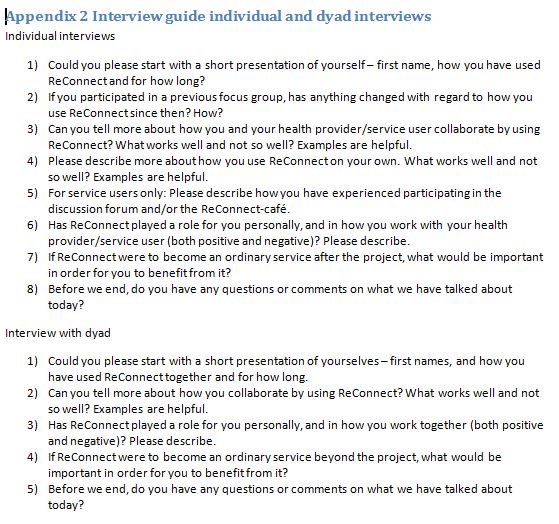

Supplement: Multimedia Appendix 2 [file mental_v4i4e54_app2.JPG]
